# Promoting research to improve maternal, neonatal, infant and adolescent health in West Africa: the role of the West African Health Organisation

**DOI:** 10.1186/s12961-017-0209-5

**Published:** 2017-07-12

**Authors:** Issiaka Sombie, Aissa Bouwayé, Yves Mongbo, Namoudou Keita, Virgil Lokossou, Ermel Johnson, Laurent Assogba, Xavier Crespin

**Affiliations:** West African Health Organisation, 175 Avenue Ouezzin Coulibaly, 01 BP 153 Bobo-Dioulasso 01, Burkina Faso

## Abstract

West Africa has adopted numerous strategies to counter maternal and infant mortality, provides national maternal and infant health programmes, and hosts many active technical and financial partners and non-governmental organisations. Despite this, maternal and infant morbidity and mortality indicators are still very high. In this commentary, internal actors and officials of the West African Health Organisation (WAHO) examine the regional organisation’s role in promoting research as a tool for strengthening maternal and infant health in West Africa.

As a specialised institution of the Economic Community of West African States (ECOWAS) responsible for health issues, WAHO’s mission is to provide the sub-region’s population with the highest possible health standards by harmonising Member States’ policies, resource pooling, and cooperation among Member States and third countries to collectively and strategically combat the region’s health problems. To achieve this, WAHO’s main intervention strategy is that of facilitation, as this encourages the generation and use of evidence to inform decision-making and reinforce practice.

WAHO’s analysis of interventions since 2000 showed that it had effected some changes in research governance, management and funding, as well as in individual and institutional capacity building, research dissemination, collaboration and exchanges between the various stakeholders. It also revealed several challenges such as process ownership, member countries’ commitment, weak individual and institutional capacity, mobilisation, and stakeholder commitment. To better strengthen evidence-based decision-making, in 2016, WAHO created a unique programme aimed at improving the production, dissemination and use of research information and results in health programme planning to ultimately improve population health.

While WAHO’s experiences to date demonstrate how a regional health institution can integrate research promotion into the fight against maternal and infant mortality, the challenges the organisation has encountered also demonstrate the importance of cohesion among actors promoting such an initiative, the importance of leadership and commitment among member country actors steering the process, and the need for collaboration and coordination among all partners in member countries and in the region.

## Background

In this commentary, we, the internal actors and officials of the West African Health Organisation (WAHO), examine our regional institution’s role in promoting research as a tool for strengthening maternal and infant health in West Africa. These reflections are meant to describe and analyse WAHO’s process for improving research, and the environment in which it is carried out, by making it easier for all national and regional stakeholders to collaborate in improving maternal and infant health.

West Africa has a number of strategies to counter maternal and infant mortality, provides national maternal and infant health programmes, and hosts many active technical and financial partners and non-governmental organisations. Despite this, maternal and infant morbidity and mortality indicators are still very high [1]. In 2015, a review sponsored by WAHO showed that West African barriers to improvements in those indicators were linked to context and health systems factors [2]. Contextual barriers included road conditions, culture, knowledge of risks and the status of women, while health system barriers included the geographic distance of health centres, services delivery organisation, the availability and ability of health services, and the quality of care. The analysis showed that these factors act together to increase maternal or infant morbidity and mortality [2], and therefore each country faces a unique challenge in reducing them. Issues surrounding governance and the ways in which decisions are made further compound these barriers. In West Africa, as is the case in many developing countries, decision-making processes are not always founded on sound evidence and are influenced by a variety of others factors, which often leads to poor policies and programmes and limits efforts to improve maternal and child health.

As a result, strengthening the fight against maternal and infant mortality must go beyond clinical training and be adapted to this wider variety of factors, including the national health research system. Doing so will contribute to improving demand, access, quality of care and satisfaction with care, along with the adaptation, implementation and scaling-up of interventions or effective strategies [3]. Key to this approach is that research be conducted and used to inform policies and practices.

Integrating research into this process, as advocated by WHO and other actors, requires knowledge of its added value and of the various actors’ skills in terms of health systems research, defined as research that focuses on governance, financial aspects, the care and services offered to the population, and the context under which they are negotiated, implemented and reformed [4]. This multidisciplinary research uses both quantitative and qualitative methods and requires stakeholder commitment [5], and can be carried out by healthcare officers, programme managers and national healthcare leaders [6]. Unfortunately, technical and organisational gaps limit this integration in developing countries, particularly in West Africa [7].

## WAHO’s approach to improving maternal health in West Africa through the promotion of research

WAHO is a specialised institution of the Economic Community of West African States (ECOWAS) responsible for health issues. It therefore operates independently of WHO, but works closely with its Regional Office for Africa. The regional organisation’s mission is to provide the sub-region’s population with the highest possible health standards through harmonising Member States’ policies, resource pooling and cooperation among Member States and third countries to collectively and strategically combat the region’s health problems. To achieve its mission and objectives, WAHO’s main intervention strategy is that of facilitation, as this encourages the generation and use of evidence to inform decision-making and reinforce practices. It has deemed it particularly important to encourage the contribution of strong partnerships that can effectively identify and address priorities. Under this strategy, WAHO’s key functions are leadership, marketing, strategic communication, policy advocacy, coordination, networking, resource mobilisation support, harmonisation support and partnership development. WAHO has deliberately adopted an approach that is based on reflection and learning by doing, which allows it to review, assess and draw lessons from its experiences so as to constantly improve its efforts as well as evolve and adapt to a variable, demanding regional context.

Quality research is a fundamental aspect of evidence-based decision-making, and promoting research as a tool for solving health problems in ECOWAS countries has played an increasingly important role for WAHO, as evidenced through its three strategic plans. While the first plan (in 2000) did not include a research-based programme, by the time the second one was written (2009–2013), research promotion became the focus of the programme on strengthening national health research systems. This was done through the application of the conceptual framework set out by Pang et al. [8], which is based on strengthening four key research functions, namely collaborative management, research funding, institutional and individual research capacity, and promoting the dissemination and use of research results. To these, WAHO added a partnership development function [9, 10]. WAHO’s analysis of interventions during this period showed that it had effected some changes in research governance, management and funding, as well as in individual and institutional capacity building, research dissemination, collaboration and exchanges between the various stakeholders. It also revealed many challenges, such as process ownership, member countries’ commitment, weak individual and institutional capacity, mobilisation, and stakeholder commitment [10–12].

To better strengthen evidence-based decision-making, research and health information were integrated into the third strategic plan (2016–2020), creating a unique programme aimed at improving the production, dissemination and use of research information and results in health programme planning to ultimately improve population health. To that end, expanding partnerships with a wide variety of stakeholders became an important factor.

While WAHO’s anchor point at the country level has always been the Ministry of Health, by integrating research into its work, the organisation has also learned to play its role alongside other stakeholders and work with networks of research centres and universities.

At the institutional level, this approach is implemented jointly by the units in charge of research, maternal and infant health, health system reinforcement, and partnership development. It is also implemented jointly with the ministries of health, research institutions, civil society, non-governmental organisations and technical partners, with the financial support of international partners and ECOWAS. These research promotion efforts are also supported by numerous regional projects [13–16].

## Preliminary results

### Strengthening stakeholder commitment

WAHO’s collaborative management efforts promote the identification of research needs and priorities, and strengthen collaboration among researchers, policymakers and other health stakeholders to foster trust and partnership. Through these efforts, WAHO has helped several countries develop and adopt their strategic documents (policies, plans and priorities) and began promoting a dialogue within countries and regions through six country workshops and one regional workshop in 2015. Participants included decision-makers, researchers, non-governmental organisation leaders, civil society actors, representatives from health professional associations, development partners, and managers of knowledge transfer platforms. During these workshops, discussions focused on the collaboration among researchers and users of research results, the inclusion of equity, gender and systemic factors, assessing stakeholders’ knowledge-transfer skills and needs, and barriers to and facilitators of evidence-based decision-making [17, 18]. The enthusiasm of stakeholders revealed that these collaborative platforms must be maintained to identify additional research priorities and needs in maternal and infant health, to engage all actors and ensure their buy-in, and to collectively identify research-supported solutions to mother and infant health problems. This collaboration should also persuade national policymakers to increase the demand for research and convince researchers to take national priorities and needs into account when designing their research.

### Strengthening the availability of evidence

WAHO funds research to increase the availability of evidence that supports informed decision-making in the field of mother and infant health. In 2014, the organisation funded a review of maternal and infant health indicators and an implementation analysis of the reproductive health programmes of 15 West African countries [19]. Among other things, this review showed that maternal and infant mortality rates had not declined far enough for many countries to achieve the 2015 Millennium Development Goals. In 2015, WAHO funded three additional reviews that discussed the issues of developing maternal and infant health programmes that consider gender and equity, systemic factors that facilitate or limit maternal and infant health improvement, and knowledge transfer. These reviews pointed to the importance of integrating, in maternal and infant health programmes, the promotion of the empowerment of women, the consideration of the needs of specific sub-groups of women and infants and of systemic and contextual factors, and the strengthening of the knowledge transfer process to support the use of evidence [20–22].

### Capacity building

To build individual and institutional capacities, WAHO grants training bursaries to young researchers and has set up a network of research institutions to support researchers’ professional development, exchanges and collaboration. WAHO is also involved in a regional capacity-building project on policy research and health systems in West Africa [15]. An inclusive regional forum was conducted in 2015 and 2016 under the University of Ghana’s leadership [23]; it analysed the strengths and weaknesses of policy and health systems research in West Africa and identified potential capacity-building actions at the regional level. Following the forum, a 5-year regional project was launched in 2016 to build the region’s capacity to steer policy and health systems research, and to disseminate results that influence maternal and infant health policies [16]. WAHO will contribute to the project through its leadership, technical means and regional network to mobilise and engage the various stakeholders needed for the project’s success.

### Strengthening knowledge transfer and partnership platforms

To promote the dissemination and use of research results, WAHO collaborates with individuals and institutions with expertise in maternal and infant health research; this includes the University of Ghana School of Public Health, the Laboratoire d'Etudes et de Recherche sur les Dynamiques Sociales et le Développement Local, United Nations institutions (WHO, UNFPA, UNICEF), and the Agence de Médecine Préventive. For example, the Agence de Médecine Préventive and WAHO have implemented technical advisory groups on immunisation in Senegal, Ivory Coast, Burkina Faso and Nigeria [24, 25]. The members of these groups are trained in research and the use of evidence and are thus competent to advise immunisation health authorities. Based on current evidence, these groups have advised country leaders on introducing new vaccines and organising vaccination programmes.

In 2015, WAHO also launched an annual regional forum in Ouagadougou, Burkina Faso, on good practices in the ECOWAS space. The aim was to support the identification, documentation, sharing and scaling-up of good health practices with the support of the United States Agency for International Development, the German Development Agency and the Canadian International Development Agency. To identify and document good practices, national training workshops in documentation methods trained more than 400 people and involved technical and financial partners in 14 countries of the ECOWAS space.

WAHO also supports the implementation of a knowledge transfer platform for the use of evidence in developing and implementing policies and programmes in maternal and individual health.

## Addressing the challenges

By commissioning these reviews, WAHO hoped to obtain the evidence-base that would allow it to play its advocacy and catalyser role, effectively engaging and mobilising all stakeholders to adopt a health systems approach towards improving maternal and infant mortality in West Africa. As such, instead of adopting imported – or travelling – models of health, country stakeholders should work together to identify the systemic and contextual elements that act as barriers, and identify solutions that are locally adaptable and applicable. This vision requires the involvement of all relevant stakeholders, but most importantly it requires them to collaborate at regional and country levels. In achieving this vision, a number of challenges need to be taken into account.

The first challenge in successfully implementing this approach involves coordinating the actors within WAHO itself. Indeed, the fact that several departments can engage in their activities without consulting the others can limit various institutional actors’ commitment and collaboration. To address this challenge, the institution’s executive has implemented a project management unit that leads quarterly planning sessions with all staff. As a result, the institution’s programmes have produced greater inter-unit collaboration, a monitoring/evaluation institutional culture, and the promotion of the use of evidence in activities in order to improve their performance.

The second challenge is persuading countries to commit to, and own, the approach given the small force of low-capacity human resources working within an environment of ceaseless management turnover. At a 2011 regional meeting, officials in charge of research management within ministries of health expressed the need to strengthen their capacities in leadership, communication, advocacy, resource mobilisation, networking, management research and monitoring/evaluation [22]. WAHO and other financial partners supported such training and continued to do so through implementation of its new strategic plan as well as through regional projects [13, 16]. In the research services field, skills in research management, leadership, advocacy, communication and networking should contribute to improving all stakeholders’ mobilisation and commitment. Additionally, platforms promoting researcher–policymaker collaboration will ensure that research is creative, innovative, multidisciplinary and guided by current needs and opportunities, the goal being to develop solutions that are based on context, as well as results that are properly used and shared [26]. Here, WAHO’s efforts to put in place a regional consultative committee composed of experienced researchers contributed to finalising quality protocols, to the successful completion of research projects and, importantly, to bringing together researchers and potential research users, including decision-makers. These efforts should be replicated.

The importance of disease-fighting research and health systems strengthening was recognised after the recent outbreak of the Ebola virus disease [27], which presented an opportunity for WAHO to persuade policymakers in the sub-region to increase their commitment to strengthening research and, especially, to make research a decision-making support tool for improving health in West Africa. To successfully face this challenge, WAHO and all partners will have to engage in meaningful collaboration.

Unfortunately, the coordination and collaboration of technical and financial partners in West African countries constitutes the third challenge. Indeed, weak coordination between various actors limits them from knowing each other’s activities and often leads to redundant activities. WAHO is working to facilitate this coordination by inviting all other stakeholders to participate in planning, implementing and assessing its programmes. WAHO has created the Partner’s Forum at the Annual Assembly of Health Ministers, which is a space for permanent dialogue among technical and financial partners. WAHO also promotes a theme-based bilateral partner collaboration framework. This permanent dialogue between WAHO and other partners also makes it possible to practise advocacy and mobilise funds towards countries’ needs and priorities, which demonstrates the institution’s leadership.

## Conclusion

In our opinion, WAHO’s experiences to date demonstrate how a regional health institution can integrate research promotion into the fight against maternal and infant mortality. At the same time, the challenges the organisation has encountered also demonstrate the importance of cohesion among actors promoting such an initiative, the importance of leadership and commitment among member country actors steering the process, and the need for collaboration and coordination among all partners in member countries and in the region. Integration that succeeds in achieving sustainable development objectives in West Africa will require cohesion, a research and monitoring/evaluation culture within WAHO, and the involvement of all implementation, technical and financial partners whose mission is to promote health-improving research. Next, WAHO and all partners should work to persuade member countries’ national leaders to steer the process towards mobilising and engaging all stakeholders; this will create an environment favourable to research, and subsequently, effective national health research systems. Finally, past experiences [10, 11] show the need to establish a learning and monitoring/evaluation system to capture all the necessary lessons and share experience with the whole community.

## References

[1] Alkema L, Chou D, Hogan D, Zhang S, Moller AB, Gemmill A, Fat DM, Boerma T, Temmerman M, Mathers C, Say L, on behalf of the United Nations Maternal Mortality Estimation Inter-Agency Group collaborators and technical advisory group (2016). Global, regional, and national levels and trends in maternal mortality between 1990 and 2015, with scenario-based projections to 2030: a systematic analysis by the UN Maternal Mortality Estimation Inter-Agency Group. Lancet.

[2] Agyepong IA, Kwamie A, Defor S, Frimpong E, Aryeetey GC, Ibrahim A. Health Systems and MNCH Outcomes in West Africa. A study of conducive and limiting health systems factors to improving mother, newborn and child health in West Africa with a focus on Ghana, Benin, Burkina Faso, Mali, Nigeria and Senegal. Bobo-Dioulasso: West African Health Organisation; 2015.

[3] Ramaswamy R, Kallam B, Kopic D, Pujic B, Owen MD (2016). Global health partnerships: building multinational collaborations to achieve lasting improvements in maternal and neonatal health. Glob Health..

[4] Hoffman SJ, Røttingen JA, Bennett S, Lavis JN, Edge JS, Frenk J. A Review of Conceptual Barriers and Opportunities Facing Health Systems Research to Inform a Strategy from the World Health Organization. Background Paper Commissioned by The Alliance for Health Policy and Systems Research to Develop the WHO Health Systems Research Strategy. The Alliance for Health Policy and Systems Research. Geneva; WHO: 2012.

[5] Lucy G (2012). Health Policy and Systems Research: A Methodology Reader. The Abridged Version, Alliance for Health Policy and Systems Research.

[6] Remme JHF, Adam T, Becerra-Posada F, D’Arcangues C, Devlin M (2010). Defining research to improve health systems. PLoS Med.

[7] Implementation Research for Control of Infectious Diseases of Poverty. Strengthening the Evidence Base for the Access and Delivery of New and Improved Tools, Strategies and Interventions. World Health Organization, UNICEF/UNDP/World Bank/WHO Special Programme for Research and Training in Tropical Diseases; 2002. http://www.who.int/tdr/publications/tdr-research-publications/access_report/en/. Accessed 16 Oct 2016.

[8] Pang T, Sadana R, Hanney S, Bhutta ZA, Hyder AA, Simon J (2003). Knowledge for better health: a conceptual framework and foundation for health research systems. Bull World Health Organ.

[9] Sombié I, Aidam J, Konaté B, Somé TD, Kambou SS (2013). The state of the research for health environment in the ministries of health of the Economic Community of the West African States (ECOWAS). Health Res Policy Syst..

[10] Aidam J, Sombié I (2016). The West African Health Organization's experience in improving the health research environment in the ECOWAS region. Health Res Policy Syst..

[11] Sombie I, Aidam J, Montorzi G. Evaluation of regional project to strengthen national health research systems in four countries in West Africa: lessons learned. Health Res Policy Syst. 2017;15(Suppl 1): doi:10.1186/s12961-017-0214-8. 10.1186/s12961-017-0214-8 PMC5516846 28722552

[12] Keita N, Lokossou V, Berthe A, Sombie I, Johnson E, Busia K. The West African experience in establishing steering committees for better collaboration between researchers and decision-makers to increase the use of health research findings. Health Res Policy Syst. 2017;15(Suppl 1): doi:10.1186/s12961-017-0216-6. 10.1186/s12961-017-0216-6 PMC5516844 28722563

[13] IDRC. Moving Maternal Newborn and Child Health Evidence into Policy in West Africa. https://www.idrc.ca/en/project/moving-maternal-newborn-and-child-health-evidence-policy-west-africa-0. Accessed 16 Oct 2016.

[14] COHRED. Strengthening Research System Development for Health in West Africa. http://www.cohred.org/westafrica/. Accessed 16 Oct 2016.

[15] IDRC. The West Africa Initiative to Strengthen Capacities through Health Systems Research. https://www.idrc.ca/en/project/west-africa-initiative-strengthen-capacities-through-health-systems-research-0. Accessed 16 Oct 2016.

[16] West and Central African Health Policy, Systems and Maternal, Newborn, Child and Adolescent health partnership. South-South capacity building and networking partnership for leadership, research and practice to support health policy, systems strengthening for improved Maternal, Newborn, Child and Adolescent Health (MNCAH) outcomes improvement in West and Central Africa. 2016.

[17] Uneke CJ, Sombie I, Keita K, Lokossou V, Johnson E, Ongolo-Zogo P (2017). An assessment of national maternal and child health policy-makers’ knowledge and capacity for evidence informed policy-making in Nigeria. Int J Health Policy Manage.

[18] Uneke CJ, Sombie I, Keita N, Lokossou V, Johnson E, Ongolo-zogo P. Improving maternal and child health policymaking process in Nigeria: an assessment of policymakers' needs, barriers and facilitators of evidence-informed policymaking. Health Res Policy Syst. 2017;15(Suppl 1): doi:10.1186/s12961-017-0217-5. 10.1186/s12961-017-0217-5 PMC5516839 28722554

[19] Organisation Ouest Africaine de la Santé (2014). Analyse de la Situation de la Santé de la Reproduction et la Planification Familiale dans l’espace CEDEAO.

[20] Larson EA (2015). Inclusion of Gender and Equity in Maternal, Newborn and Child Health Services in West Africa: A Literature Review of Programming the West African Health Organization.

[21] Agyepong IA, Kwamie A, Frimpong E, Defor S, Aryeetey GC, Abdallah I, Virgil Lokossou V, Sombie I. Spanning maternal newborn and child health (MNCH) and health systems (HS) research boundaries: conducive and limiting health systems factors to improving MNCH outcomes in West Africa. Health Res Policy Syst. 2017;15(Suppl 1): doi10.1186/s12961-017-0212-x. 10.1186/s12961-017-0212-x PMC5516848 28722556

[22] Pierre OZ (2016). Analyse de la Situation sur le Transfert de Connaissance au Profit de la Santé Maternelle et Néonatale en Afrique de l’Ouest.

[23] Defor S, Kwamie A, Sombie I. Strengthening Health Policy and Systems Research Capacity in West Africa: Lessons and Experiences from a Collaborative Sub-regional Effort. Symposium #G1. 22ème Conférence Canadienne sur la Santé Mondiale. Renforcement des Capacités en Santé Mondiale: Recherche et Pratiques. 5–7 November 2015, Montréal.

[24] Adjagba A, Senouci K, Biellik R, Batmunkh N, Faye PC, Durupt A, Gessner BD, da Silva A (2015). Supporting countries in establishing and strengthening NITAGs: lessons learned from 5 years of the SIVAC initiative. Vaccine..

[25] NITAG Resource Center. Les groupes techniques consultatifs de la vaccination. http://www.nitagresource.org/fr/qui-sommes-nous#nrc-map. Accessed 03 Sep 2016.

[26] Sipido K, Degos L, Frackowiak R, Ganten D, Hofstraat H, Horvath I, Luyten F, Manns M, Oertel W, Zima T (2016). Scientific Panel for Health: better research for better health. Lancet..

[27] Massaquoi MBF, Kennedy SB, Tegli JK, Bolay FK, Kateh KN (2016). Fostering collaboration on post-Ebola clinical research in Liberia. Lancet..

